# Socioeconomic inequalities in cardiometabolic control in patients with type 2 diabetes

**DOI:** 10.1186/s12889-018-5269-0

**Published:** 2018-03-27

**Authors:** Berta Ibáñez, Arkaitz Galbete, María José Goñi, Luis Forga, Laura Arnedo, Felipe Aizpuru, Julián Librero, Oscar Lecea, Koldo Cambra

**Affiliations:** 1 Navarrabiomed-CHN-UPNA, C/ Irunlarrea 3. Recinto CHN, 31008 Pamplona, Spain; 2 Red de Investigación en Servicios de Salud en Enfermedades Crónicas (REDISSEC), Pamplona, Spain; 3 IdiSNA, Pamplona, Spain; 4 Servicio de Endocrinología y Nutrición, Complejo Hospitalario de Navarra, Servicio Navarro de Salud-Osasunbidea, Pamplona, Spain; 5 Instituto de Estadística de Navarra, Pamplona, Spain; 6 Hospital de Txagorritxu, Servicio Vasco de Salud-Osakidetza, Gasteiz, Vitoria, Spain; 7 Gerencia de Atención Primaria, Servicio Navarro de Salud-Osasunbidea, Pamplona, Spain; 8 Instituto de Salud Pública y Laboral de Navarra, Pamplona, Spain

**Keywords:** Socioeconomic status, Socioeconomic inequalities, Diabetes mellitus, Diabetes care, Cardiometabolic risk factors, Cardiovascular control, Metabolic control, Glycated haemoglobin

## Abstract

**Background:**

The aim of this study was to determine if the achievement of control targets in patients with type 2 diabetes was associated with personal socioeconomic factors and if these associations were sex-dependent.

**Methods:**

This cross-sectional, population-based study was conducted in Spain. Glycated haemoglobin (HbA1c) level and other clinical parameters were obtained from electronic primary care records (*n* = 32,638 cases). Socioeconomic status was determined using education level and yearly income. Among patients, having their HbA1c level checked during the previous year was considered as an indirect measure of the process of care, whereas tobacco use and clinical parameters such as HbA1c, low-density lipoprotein cholesterol (LDL-c) and blood pressure (BP) were considered intermediate control outcomes. General linear mixed effect models were used to assess associations.

**Results:**

The achievement of metabolic and cardiovascular control targets in patients with type 2 diabetes was associated with educational level and income, and socioeconomic gradients differed by sex. The probability of having had an HbA1c test performed in the previous year was higher in patients with lower education levels. Patients in the lowest income and education level categories were less likely to have reached the recommended HbA1c level. Males in the lowest education level categories were less likely to be non-smokers or to have achieved the blood pressure targets. In contrast, patients within the low income categories had a higher probability of reaching the recommended LDL-c level.

**Conclusions:**

Our results suggest the presence of socioeconomic inequalities in the achievement of cardiovascular and metabolic control that differed in direction and magnitude depending on the measured outcome and sex of the patient. These findings may help health professionals focus on high-risk individuals to decrease health inequalities.

## Background

Type 2 diabetes prevalence has experienced a rapid global increase in recent decades, reaching 8.8% in people over the age of 20 years in 2017, and is expected to continue rising, with a projected prevalence of 10.4% by 2040 [1]. This chronic metabolic condition has been associated with high morbidity and disability, and confers an approximately two-fold greater risk of cardiovascular disease independent of other conventional risk factors [2]. This implies an exorbitant cost to both patients and society that accounted for approximately 12.5% of total health expenditures worldwide in 2017 [1]. Optimal disease management has been recognized to lead to fewer complications, [3, 4]. However, the achievement of cardiometabolic risk factor control in diabetic patients remains challenging, and the identification of factors associated with optimal control has become a key issue in recent literature [5–7].

Social determinants of health, defined as economic and social conditions that influence the health of people and communities, play an important role in type 2 diabetes. It has been well recognized that having a low socioeconomic status (SES), commonly measured in terms of education, income, occupational social class or financial wealth, may be associated with a higher incidence and prevalence of diabetes [8–10]. In patients with this pathological condition, low SES has been shown to be associated with mortality [11–13]. Further, according to a recent systematic literature review [14], low individual SES and regional deprivation were found to be frequently associated with worse process and intermediate outcome indicators in these patients. Nevertheless, some studies did not find these associations with intermediate outcomes [15, 16], others found associations for only some of them, [17–20] and only a few studies assessed if social gradients in cardiovascular risk factors differed by gender. Although the exact mechanisms or pathways linking socioeconomic status to health in persons with diabetes mellitus are unknown, a conceptual framework by Brown et al. [21], which was empirically modified and validated by Walker et al. [22], attempted to elucidate these associations. These authors suggested that individual features as well as contextual factors, such as communities and health care organizations, influence this association. They hypothesized that social determinants were directly associated with diabetes-related health outcomes, in addition to being associated with self-care, access to care and processes of care, which are also associated with these health outcomes.

Health inequality varies across regions and countries, and it is not merely a reflection of income inequality [23]. Spain has relatively low - although still significant - income-related health inequalities compared to other European countries [23]. Citizens are covered by the National Health Services (NHS) of Spain, a decentralized structure administered by each autonomous government that provides practically universal healthcare financed by general taxes, and health services, such as hospitalizations and diagnostic procedures, are free of charge for all citizens. A fraction of medication costs is paid for by patients within a cost-sharing scheme established in Spain in July 2012 that is based on their employment and income status, with some regional variations [24]. In spite of the relatively low health inequality in Spain, there exists a substantial contribution of regional health disparities on the socioeconomic inequalities of the country [23, 25], with northern regions such as Basque Country, Navarre and La Rioja presenting the lowest degree of these inequalities [25]. The monitoring of health inequalities has been officially included in the political agenda in Spain [26], but research assessing the impact of SES on intermediate outcomes for chronic diseases in the country is still scarce [27].

This work aimed to assess the extent to which SES, measured at the individual level, is a determinant of the achievement of control of metabolic/cardiovascular risk factors in patients with type 2 diabetes in one of the regions of Spain with the lowest health inequality, and whether this association differs between women and men.

## Methods

This population-based cross-sectional study was conducted in Navarre, an autonomous region in northern Spain with more than 600,000 inhabitants. In this region, citizens are covered by the Regional Health Service of Navarre – Osasunbidea, which is part of the NHS of Spain. They are commonly served by one primary care centre with a stable team of general practitioners, nurses, paediatricians and other healthcare workers, and only 3.2% of the population has private or mixed health insurance [28].

Two sources of information were available for use in this study. The Primary Care Electronic Medical Record System of the Regional Health Service of Navarre, named Atenea, was used to identify patients with diabetes and to obtain their demographic and clinical data. This system was established in the early 2000s and has been comprehensively used by all health professionals since 2008. It contains diagnosis, clinical, lifestyle, laboratory results, and prescription data. Additionally, the 2013 population register was used to obtain the educational level of the patients. All patients over 20 years of age registered in Atenea with a diagnosis of type 2 diabetes (code T90 within the International Classification of Primary Care) on May 15, 2014 were included in this study. The extraction of data from Atenea records was conducted by the Department of Informatics, Telecommunication and Public Innovation of the Government of Navarre (DGITIP) using the SQL Server Integration Services Platform, and was linked with the population register by the Institute of Statistics of Navarre. Of a total of 33,346 patients with an active code of type 2 diagnosis, 693 (2.1%) had no clinical history record number or individual identification code, and 15 were excluded for being < 20 years old, resulting in a working sample of 32,638 patients. Regarding population data, of the total of 33,346 patients, 31,792 (95.3%) were linked with the individual patient identification code, 1044 (3.1%) with the identity card and 25 (0.1%) using name and birth data, and the remaining 485 (1.5%) could not be found in the population register and were missing for the educational level variable. The linked dataset with 32,638 patients was anonymized before transfer to the researchers.

Data from laboratory test results, including the glycated haemoglobin (HbA1c) level; systolic and diastolic blood pressure (SBP and DBP); LDL- and HDL-cholesterol and triglyceride levels; and demographic and clinical data, such as weight, height, body mass index (BMI), smoking status (current smoker versus not current smoker), medication use, date of registration in the information system, date of diabetic onset and birth date, were included in this study. We used the latest data available within the 15 months prior to the data extraction date. Having at least one measurement of HbA1c within the previous year was considered an indirect indicator of the process of care [11]. To assess the achievement of control objectives, the following thresholds were used: HbA1c level < 7% (53 mmol/mol), blood pressure ≤ 140/90 mmHg, LDL-cholesterol level ≤ 100 mg/dl and no tobacco consumption.

We considered education and copayment level, the latter used as a proxy of income, as indicators of socioeconomic status. Education level was aggregated according to four levels: unfinished primary education, primary education, secondary education (high school) and college (and above) education. The copayment level, the coinsurance status of the patients that determines the percentage of the price of the drug to be paid by the patient, is assigned to each insured person based on his/her annual income and pensioner status (pensioner or non-pensioner). Three categories were created for this study: yearly income < 18,000 €, yearly income ≥18,000 €, and those who were excluded or exempted for payment, which included people who were unemployed without benefits; were receiving the minimum subsistence income; had been diagnosed with a toxic syndrome, certain disabilities, work-related accidents or occupational disease; or were pensioners with non-contributory retirement pensions.

### Statistical analysis

Patient characteristics and the achievement of control targets were summarized using frequencies and percentages. To examine the relationship between educational level and each of the control target criteria, a mixed effects logistic regression model with complete cases for each target criteria was fitted. The models included educational level as exposure variable age (continuous) and sex as control variables, and basic health zone as random effects, to account for any variability between zones. The same model structure was used to examine the relationship between income level and each control target criterion. The highest level of study and the highest level of income were considered the reference categories. Specific models for males and females were also fitted to assess gender-specific social gradients, and they were used for interpretation purposes when interactions between sex and SES variables were significant at *p* < 0.05. Complementary, specific models for pensioners and non-pensioners were fitted, and the results of these models are displayed as footnotes only for the two models for which the interaction between SES and pensioner status was significant. Variable significance was determined using the likelihood ratio test, and adjusted odds ratios with 95% confidence intervals were derived for each fitted model. All statistical analyses were performed using the statistical package R, version 3.2.0.

## Results

In total, 32,638 patients over 20 years of age with type 2 diabetes were registered in the Navarre Regional Health Service, and 679 of them (2%) had been diagnosed within the last year. The sociodemographic characteristics of the patients and the proportion of patients not achieving control targets are given in Table 1. The percentage of non-missing data was over 95% for all sociodemographic characteristics and over 70% for all indicators of disease control considered except for tobacco use, which was 38%. Of the patients, 56% were men, 70% were aged 65 years or older and 73% were pensioners. Most of the patients belonged to the lowest income categories of copayment (69% had income < 18,000 € and 4% were excluded or exempted for payment), and more than half (53%) had a primary level of education.

**Table 1:** Descriptive characteristics of the sample and fulfilment of target criteria

Characteristics	*N* available^a^	Control previous Year (%)^b^	Unachieved targets (%)^c^			
			HbA1c > 7%	LDL > 100 mg/dl	BP > 140/90 mmHg	Current smoker
	(*n* = 32,638)	(*n* = 32,638)	(*n* = 23,094)	(*n* = 23,891)	(*n* = 24,595)	(*n* = 12,413)
Age	32,638(100%)	70.8	39.9	59.5	30.1	16.0
< 65 years	10,803(33.1%)	64.3	41.5	64.6	26.5	30.9
≥ 65 years	21,835(66.9%)	73.9	39.2	57.4	31.5	9.9
Sex	32,206(98.7%)	71.2	39.9	59.5	30.1	16.0
Men	18,188(56.5%)	70.9	38.8	55.3	30.0	21.3
Women	14,018(43.5%)	71.6	41.2	64.8	30.3	8.8
Pensioner situation	32,638(100%)	70.8	39.9	59.5	30.1	16.0
Non-pensioner	8838 (27.1%)	63.1	41.7	66.2	28.0	29.6
Pensioner	23,800 (72.9%)	73.7	39.3	57.4	30.7	11.9
Education level	32,060(98.2%)	71.3	39.8	59.5	30.1	16.0
College education	1655(5.2%)	63.1	33.2	59.5	28.8	17.0
Secondary education	3056(9.5%)	65.8	36.7	60.8	28.6	24.7
Primary education	16,869(52.6%)	72.2	40.0	60.1	30.0	17.0
Without primary education	10,480(32.7%)	72.6	41.3	58.2	30.7	11.9
Income	31,709 (97.2%)	70.9	39.8	59.5	30.1	16.0
≥ 18,000 €	8372 (26.4%)	72.5	36.5	60.6	29.4	19.6
< 18,000 €	21,95,269.2%)	70.4	41.0	59.0	30.3	14.2
Excluded/exempted	1385 (4.4%)	70.6	42.2	61.6	31.5	23.2

^a^*N* available: Number of patients and percentage of data available for each variable and number and percentage of patients in each category

^b^Control previous year: Percentage of patients with HbA1c data within the past 15 months

^c^Unachieved targets: Percentage of patients within each category that did not reach the current local control targets for each risk factor

Guideline compliance results showed that the target achievement varied based on the assessed criterion. The criterion of having had ‘at least one basic laboratory test including HbA1c levels performed during the previous year’, as an indicator of adequate adherence to clinical practice, was achieved in 71% of the diabetic patients and was more frequently identified in patients over the age of 65 years (74%), pensioners (74%), and patients with lower education levels (73%). Failure to achieve control targets was observed in a high proportion of patients, as demonstrated by the LDL level > 100 mg/dl (59%), HbA1c level > 7% (40%) and BP > 140/90 mmHg criteria (30%). Approximately 16% of the patients were registered as smokers, and this proportion increased to 21% in men and 31% in those < 65 years old. An assessment of sex and age differences in the achievement of control targets in this population can be found in Cambra et al. [29].

### Associations between SES indicators and regular HbA1c measurement

Education level was found to be associated with the evaluated medical process measure (see first column in Table 2). Patients with lower educational level were more frequently identified as having had a basic laboratory test conducted during the previous year, and this gradient was significantly steeper in females than in males. Compared to women with a university education, women with a lower education level had two-fold greater odds of testing; whereas in men, the odds were 1.2-fold greater.

**Table 2:** Age- and sex-adjusted and sex-specific logistic regression model results for the failure to achieve control targets by educational level

	Control previous year^a^		Unachieved targets^b^							
			HbA1c > 7%		LDL > 100 mg/dl		BP > 140/90 mmHg		Current smoker	
	OR (95% CI)	*P*	OR (95% CI)	*p*	OR (95% CI)	*p*	OR (95% CI)	*p*	OR (95% CI)	*p*
Total sample										
Sex										
Men	Reference		Reference		Reference		Reference		Reference	
Women	0.95(0.91,1.00)	0.068	1.11(1.05,1.17)	< 0.001	1.62(1.54,1.72)	< 0.001	0.96(0.91,1.02)	0.219	0.43(0.38,0.49)	< 0.001
Education level										
University	Reference		Reference		Reference		Reference		Reference	
Secondary	1.20(1.06,1.37)	< 0.001^c^	1.12(0.96,1.32)	< 0.001^c^	0.99(0.85,1.16)	0.576	1.09(0.92,1.29)	0.753^c^	1.37(1.05,1.80)	0.008^c^
Primary	1.52(1.36,1.70)		1.27(1.11,1.46)		0.99(0.87,1.13)		1.08(0.94,1.26)		1.32(1.05,1.68)	
Without	1.47(1.30,1.65)		1.34(1.16,1.55)		0.95(0.83,1.09)		1.08(0.93,1.26)		1.12(0.87,1.45)	
Males										
University	Reference		Reference		Reference		Reference		Reference	
Secondary	1.18(1.01,1.38)	< 0.001	1.00(0.83,1.20)	0.004	0.99(0.83,1.19)	0.976	1.22(1.00,1.50)	0.085	1.48(1.09,2.01)	0.052
Primary	1.38(1.20,1.58)		1.10(0.94,1.29)		1.02(0.87,1.19)		1.25(1.05,1.49)		1.44(1.10,1.90)	
Without	1.24(1.07,1.43)		1.23(1.04,1.46)		1.01(0.86,1.20)		1.19(0.99,1.44)		1.36(1.03,1.82)	
Females										
University	Reference		Reference		Reference		Reference		Reference	
Secondary	1.20(0.94,1.52)	< 0.001	1.59(1.15,2.23)	< 0.001	0.94(0.68,1.31)	0.079	0.74(0.53,1.02)	0.153	1.16(0.86,1.56)	0.001
Primary	1.91(1.56,2.33)		2.00(1.52,2.66)		0.85(0.65,1,11)		0.74(0.57,0.98)		1.00(0.77,1.28)	
Without	1.99(1.62,2.45)		1.98(1.50,2.65)		0.78(0.59,1.03)		0.78(0.59,1.02)		0.64(0.49,0.83)	

All models were adjusted by age (continuous) and basic health zone (random effect), resulting in significant *p*-values for both in all cases

^a^Control previous year: Response variable is 'To have HbA1c data within the past 15 months'

^b^Unachieved targets: Response variable for each risk factor is 'Not to have reached current local targets'

^c^Interaction between educational level and c sex was significant

Income was also associated with the probability of having had a test performed within the past year; however, significantly different gradients were identified in non-pensioners and pensioners and in men and women (see first column in Table 3 and footnote c). In female non-pensioners, those excluded from payment and those in the low-income category had significantly greater odds of having had a test performed than those in the high income category (OR = 1.71 and OR = 1.37 respectively *p* < 0.001). In male pensioners, however, an inverse gradient was observed, and patients in the lowest income categories were significantly less likely to have had a test performed.

**Table 3:** Age- and sex-adjusted and sex-specific logistic regression model results for the failure to achieve control targets by income category

	Control previous year^a^		Unachieved targets^b^							
			HbA1c > 7%		LDL > 100 mg/dl		BP > 140/90 mmHg		Tobacco use	
	OR (95% CI)	*p*	OR (95% CI)	*p*	OR (95% CI)	*p*	OR (95% CI)	*p*	OR (95% CI)	*p*
Total										
≥ 18.000 €	Reference		Reference		Reference		Reference		Reference	
< 18.000 €	0.89(0.83,0.94)	< 0.001^c^	1.19(1.12,1.27)	< 0.001	0.90(0.85,0.96)	0.004	1.00(0.94,1.08)	0.119	0.98(0.88,1.10)	0.375^d^
Excluded	1.01(0.89,1.15)		1.19(1.04,1.37)		0.86(0.74,0.99)		1.16(1.00,1.34)		1.17(0.91,1.50)	
Males										
≥ 18.000 €	Reference		Reference							
< 18.000 €	0.80(0.75,0.86)	< 0.001	1.22(1.13,1.32)	< 0.001	0.89(0.82,0.96)	< 0.001	0.99(0.91,1.07)	0.126	1.02(0.90,1.16)	0.385
Excluded	0.93(0.77,1.12)		1.27(1.03,1.56)		0.73(0.59,0.90)		1.24(0.99,1.54)		1.25(0.91,1.72)	
Females										
≥ 18.000 €	Reference		Reference							
< 18.000 €	1.09(0.98, 1.22)	0.086	1.14(1.01,1.28)	0.102	0.93(0.82,1.05)	0.425	1.05(0.93,1.19)	0.442	0.82(0.64,1.08)	0.339
Excluded	1.22(1.01,1.47)		1.11(0.91,1.36)		1.00(0.81,1.23)		1.15(0.93,1.41)		0.92(0.60,1.40)	

All models were adjusted by age (continuous) and basic health zone (random effect), resulting significant p-values for both in all cases

^a^Control previous year: Response variable is 'To have HbA1c data within the past 15 months'

^b^Unachieved targets: Response variable for each risk factor is 'Not to have reached current local targets'

^c^Interaction between income and sex was significant, and interaction between income and pensioner status was significant:

OR for non-pensioners: in males, category ‘< 18,000 €’ 0.93(0.81, 1.04); category excluded 1.19(0.96, 1.48); in females, category ‘< 18,000 €’ 1.37(1.11, 1.69), excluded 1.71(1.28, 2.29)

OR for pensioners: in males, category ‘< 18,000 €’ 0.76(0.70, 0.84); category excluded 0.58(0.39, 0.88); in females, category ‘< 18,000 €’ 1.07(0.94, 1.21), excluded 1.04(0.80, 1.35)

^d^Interaction between income and pensioner status significant. OR for non pensioners: category ‘< 18.000 €’ 1.10(0.91, 1.33), excluded 1.01(0.75,1.35), OR for pensioners: category ‘< 18,000 €’ 0.97(0.84, 1.13), excluded 1.85(1.11, 2.96)

### Associations between SES indicators and glycaemic control

Patients with lower education levels had higher odds of not meeting HbA1c targets, and the gradient was steeper in women. For women, the odds of noncompliance were approximately twice as high for those with primary education or without education than for those with a university education, whereas in males, the odds were close to 1.2 times as high (see Table 2).

The association between income and glycaemic control was significant, and the gradients did not differ significantly by sex or pensioner status. Greater odds of noncompliance were identified in patients within the low-income and excluded categories than those within the high-income category, with an estimated magnitude of approximately 1.20 in both cases (Table 3).

### Associations between SES indicators and cardiovascular risk factors

Education level was not significantly associated with LDL compliance; however, education was significantly associated with tobacco use and marginally associated with BP targets, specifically in males. Compared to male patients with a university education, those with lower levels of education had approximately 20% greater odds of not meeting BP targets and approximately 40% greater odds of being smokers. In contrast, female patients without a university education had approximately 36% lower odds of being tobacco users than females with a university education (Table 2).

Patients in the low-income categories were more likely to achieve the recommended LDL targets than those in the high-income category. Patients in the excluded category had 16% greater odds of not meeting BP targets, and pensioner patients in this category had two-fold greater odds of being smokers (Table 3).

## Discussion

In this large population-based study including people with type 2 diabetes, we found that socioeconomic status was associated with metabolic and cardiovascular control target achievement and that socioeconomic gradients were not the same in men and women for all control factors. Patients with low SES were more likely to have had an HbA1c test performed in the previous year, except for pensioner males, and were less likely to have reached the recommended HbA1c level. Regarding cardiovascular risk factors, males in the lowest SES categories had lower odds of having reached the BP and tobacco targets, which was in contrast with the LDL results because patients in the low SES categories were more likely to have achieved the LDL target.

The higher probability of having had at least one measurement of HbA1c in the previous year in patients within the lowest education levels was identified in both sexes, with a more marked gradient observed in women. These results are in agreement with other studies conducted in Spain [30] and other European regions [31], but not with others conducted further afield [14, 32]. Our findings are probably related to the fact that people in the lowest socioeconomic positions in Spain are more likely to visit general practitioners than those in the highest position [30, 33], even when taking into account need for care [34]. Additionally, citizens in high socioeconomic positions make more frequent use of private services, [33] and could therefore have had the HbA1c test done without being registered in the Atenea records. However, it does not completely explain our findings since, in Navarre, the proportion of people using private or mixed health insurance is only 3.2% [28], which is much lower than the differential in the percentage of patients with the test done across educational levels (9.5%). Interestingly, this apparent protective effect identified in patients with low SES was not observed in male pensioners, who were in fact less likely to have had the test conducted. To interpret this result, we should take into account the fact that patients with diabetes-related complications could be referred to specialized services for follow-up, and could therefore have their HbA1c measured out of the primary care setting, without being registered in Atenea. Referrals to specialized services could have been more frequent in patients with low SES, as they have a lower degree of HbA1c control, which could have induced an overestimation of this reverse gradient. Given the relevance of equity in access on health outcomes [21], this question needs further attention.

Our finding that individuals with the lowest income and education levels were less likely to have reached the recommended HbA1c control targets is in line with previous research. A review on the topic carried out by Grintsova et al. [14] showed that, out of 18 studies examining the association between HbA1c and SES (10 at the area level and 8 at the individual level), 11 studies identified a significant association, 6 studies identified a statistically non-significant association in the same direction, and only one study did not show any difference between deprivation groups. In our study, the gradient associated with education level was steeper in women. These result seem to correspond with the results of other European studies, such as a study carried out in Sweden [35], in which the crude risk of reaching HbA1c target levels in patients with higher education levels was also twice that of patients with lower educational levels in women and approximately 1.5 times greater in men. A recent review of sex and gender differences in the risk and complications of type 2 diabetes mellitus [36] suggested that the stronger associations between SES indicators, abdominal obesity and physical activity observed in women than in men may be behind the apparently greater sensitivity to socio-contextual predictors often observed in women; however, more studies are needed to clarify this complex issue.

The association between SES and achievement of BP targets observed in this study was marginal. The higher level of optimal BP control observed in patients with high study levels, especially in males, is in agreement with the results of studies carried out in Europe [37] and the US [20] in patients with diabetes, and is also in line with one study conducted in Spain in the general population [27]. However, the results have not been conclusive, neither in our work nor in the aforementioned review [14], where only one of the five studies evaluating this association in patients with diabetes found a significant association between BP and SES.

Our results identifying an association between tobacco consumption and low education levels in males also correspond with previous studies specific to patients with diabetes [20, 38] and are concordant with results observed from the general population [39]. The fact that women with diabetes and low education levels were less frequently tobacco users was also identified in neighbouring regions, such as the Basque country [30], but not in other European countries such as Scotland, where the risk of smoking in patients with low SES was higher regardless of sex [38]. To reduce this social inequality, targeted preventive and cessation programmes directed more strongly at people belonging to lower social classes are needed [39], since more general policies regulating tobacco consumption, such as Spanish Law 28/2005, have been shown to be more effective in individuals belonging to higher social classes [40].

We did not identify an association between educational level and lipid control; however, low income was unexpectedly associated with better LDL results. This somewhat contrasts with the results of other research studies included in the review by Grintsova et al. [14], which found no significant association between SES and lipid control in seven of the nine studies conducted at the individual- level and a significant but inverse association in the other two studies. We identified only two other studies including patients with diabetes with results in line with those of our study: a study carried out in the US found that patients with high education levels were less likely to have good cholesterol control [20], and a recent study also conducted in the US [41] suggested that employment was associated with higher LDL levels. We could not determine the reasons for this finding; however, it deserves to be noted that our region is fairly rural and has strong links to agriculture and a Mediterranean diet that is widespread in its adoption. Considering that adherence to a Mediterranean dietary pattern is not affected by educational status in the general population in Spain [42] and that daily cholesterol intake is higher in those with higher educational levels in similar Mediterranean regions of Spain [43], our results may not be unexpected.

Our work corroborates the influence of socioeconomic position on intermediate health outcomes in patients with diabetes, even in a region with one of the lowest health inequality levels of Spain [25], a country that, in turn, has relatively low health inequality compared to other European countries [23]. Apart from SES-related individual characteristics that could favour results in patients with higher SES, such as healthy diet [44, 45], physical activity [46], medication adherence [47], social support [44, 46] or self-efficacy [48], there are also contextual factors, such as the way health care institutions are organized and the health policies they adopt, that could differentially affect patients according to their SES level [21]. This differential influence may occur even in health care systems that provide universal coverage, such as the Spanish health system, where some organizational barriers, such as copayments, may pose greater obstacles for people of lower SES [21, 24]. Hence, accounting for socioeconomic status is recommended when designing action plans aiming at reducing adverse health outcomes, since these programmes are likely to be more effective if they target those with low socioeconomic status, as they are at higher risk of worse health outcomes. In an era where the need for more individualized management for patients with diabetes has been jointly stated by the European Association for the Study of Diabetes and the American Diabetes Association to overcome the highly standardized diabetes protocols [49], the incorporation of socioeconomic dimensions in addition to health variables could also help to improve health results.

The main strength of this work was that it used data from the real medical practice of all patients diagnosed in this region and encompassed all age ranges, comorbidities and social conditions identified in the population attended by the Regional Health Service, which accounts for 96% of the entire population in the region. Another strength is that we were able to link clinical data from individual patients to demographic and socioeconomic data from the population registers. Data on education level and copayment level were highly complete, with less than 2% and 3% of missing data respectively. These two individual-level measures of SES are more robust than other indirect or aggregated measures. Among the limitations, it is possible for a bias resulting from the use of existing electronic clinical records to have impacted this study, which may be particularly important in variables for which data completeness was more dependent on the physicians’ reporting procedures, such as tobacco use. Second, other important factors, such as physical inactivity, body mass index or time since diagnosis were not been included in this analysis because they were insufficiently reported in the clinical records. Finally, the main source of information was the primary care electronic medical record system; thus, data from patients who have moved away from Navarre, data from patients followed in specialist services and data from patients receiving care in private health centres were not included in this study, which could have specially influenced on the results regarding the probability of having a regular HbA1c measurement.

## Conclusions

In summary, we found the presence of socioeconomic inequalities in the achievement of control targets in type 2 diabetic patients and identified population groups that should be targeted by interventions developed to reduce these health inequalities and potentially improve the rates of achievement of these control targets. These interventions should take into account the multifaceted nature of the influence of socioeconomic factors on glycaemic control and other cardiovascular risk factors and the differential gradients observed between males and females and between pensioners and non-pensioners. It would also be recommended to develop tools to incorporate information regarding socioeconomic status and other social determinants into the electronic clinical records to enable healthcare professionals to take them into consideration during daily clinical practice.

## Abbreviations

BHZ: Basic health zone
BMI: Body mass index
BP: Blood pressure
DBP: Diastolic blood pressure
HbA1c: Glycated haemoglobin
HDL: High density lipoprotein
LDL: Low density lipoprotein
NHS: National Health Services
SBP: Systolic blood pressure
SES: Socioeconomic status
